# Premium ethylcellulose polymer based architectures at work in drug delivery

**DOI:** 10.1016/j.ijpx.2019.100023

**Published:** 2019-07-08

**Authors:** Oluwatoyin A. Adeleke

**Affiliations:** Immunobiology Section, Laboratory of Parasitic Diseases, National Institute of Allergy and Infectious Diseases, National Institute of Health, US Department of Health and Human Services, Bethesda, MD 20892, USA; Division of Pharmaceutical Sciences, School of Pharmacy, Sefako Makgatho Health Sciences University, Pretoria 0208, South Africa

**Keywords:** Hydrophobic biomaterial, Cellulose derivative, Polymeric drug delivery, Pharmaceutical excipient, Polymeric biomaterial

## Abstract

Premium ethylcellulose polymers are hydrophobic cellulose ether based biomaterials widely employed as biocompatible templates for the design of novel drug delivery systems. They are classified as United States Food and Drug Administration Generally-Recognized-As-Safe chemical substances and have been extensively utilized within the biomedical and pharmaceutical industries for over half a century. They have so far demonstrated the potential to modulate and improve the physiological performance of bioactives leading to the desired enhanced prophylactic and therapeutic outcomes. This review therefore presents a scholarly survey of inter-disciplinary developments focused on the functionalities of ethylcellulose polymers as biomaterials useful for the design of smart delivery architectures for relevant pharmacotherapeutic biomedical applications. Emphasis was placed on evaluating scientific resources related to recent advancements and future directions associated with its applications as delivery systems for drugs and biologics within the past decade thus complementing other specialized reviews showcasing the theme.

## Graphical abstract

**Figure:**
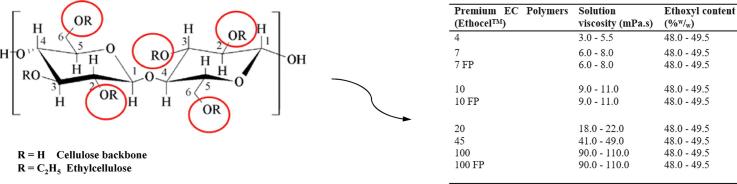

## 1 Introduction

Drug-related investigations have long shifted from just the discovery and synthesis of new and potent molecules towards the development of effective drug formulations or delivery systems which are actually more crucial and directly related to the desired pharmacotherapeutic efficacy within the body (Fenton et al., 2018). Besides, the actual design of the delivery system and its mode of application significantly impact in vivo pharmacokinetics, cell level uptake, tissue distribution, metabolism and excretion, clearance and overall adverse effects which all contribute towards the overall efficacy of the bioactive agent (Fenton et al., 2018, Jaipakdee et al., 2018, Joseph et al., 2017). Despite continuous research advancements, optimal delivery of drugs remains a key challenge especially with the limitation associated with understanding biological barriers which led to a significant increase in research effort focused on the fabrication of novel biomaterials that can positively transform drug delivery efforts (Fenton, et al., 2018). A biomaterial can be referred to as a non-drug matter, surface or construct that is capable of interacting with biological systems (Pavlovic, 2015). They can be derived from natural sources or synthesized and are often composed of various materials such as polymers, metals, glass, ceramic etc. and are required to be biocompatible but not necessarily biodegradable depending on their respective applications (Hélary et al., 2015, Lee et al., 2014, Pavlovic, 2015).

Polymeric biomaterials have significantly impacted our day to day living. They play major roles in the food and beverages, clothing, automobile, packaging, water purification, sporting etc. industries (Baldino et al., 2017, Joseph et al., 2017, Radziszewski and Saga, 2017). They are macromolecules that have been extensively explored within the pharmaceutical and medical spheres as design templates for delivery systems containing drugs or biologicals. This is attributable to their exceptionally superior physical, chemical and mechanical properties that have completely changed the face of disease prophylaxis and therapy (Fenton et al., 2018, Joseph et al., 2017, Pavlovic, 2015). They have been employed in the development of a wide selection of smart delivery architectures such as nano/micro structures, liposomes, lipid-polymer platforms, stimulus responsive systems, implants, colloids, hydrogels, prolonged release systems, targeted delivery templates capable of eliciting varying functionalities and properties that make them desirable for biomedical applications. With bioactive delivery systems based on polymeric biomaterials, it is often possible to control/tailor bioactive release kinetics to meet specific therapeutic needs as opposed to conventional formulations which are usually less flexible, difficult to regulate and lack specificity (Dow, 2014, Dow, 2016, Joseph et al., 2017). These continuous advancements have directly impacted the significant growth in the number of polymer-based drug products that are under clinical trial investigations or been approved by regulatory bodies (Fenton et al., 2018, Joseph et al., 2017).

Ethylcellulose is a partly O-ethylated cellulose ether polymeric biomaterial, typically hydrophobic in nature and widely applied within the biomedical and pharmaceutical industry for over half a century (Rekhi and Jambhekar, 1995, Mehta et al., 2016, United States, 2018). It is commercially produced with different viscosity grades keeping the variant ethoxy content within strict specification range for all types (Mehta et al., 2016). The various ethylcellulose types, usually distinguished by their nominal viscosities, molecular weights and ethoxy substitutions, are collectively referred to as “Premium Ethylcellulose Polymers”, commercially marketed under the trade name Ethocel^TM^ (Dow, 2014, Dow, 2016, Mehta et al., 2016) and may contain suitable antioxidants (United States Pharmacopeia, 2018). Ethocel^TM^ polymers are typically tasteless, free-flowing, white to light tan-colored powder, odorless, non-caloric, and inert within the physiological environment (Rowe et al., 2009, Dow, 2016). Despite the continued trend towards the use of aqueous systems, the use of the ethylcellulose polymers is still growing. Ethylcellulose is biocompatible and approved by the United States Food and Drug Administration as a Generally-Recognized-As-Safe chemical substance that has demonstrated the potential to modulate and improve the physiological performance of bioactives through its frequent applications in controlled release solid dosage forms, granulation binders, film formers, coating materials, taste masking, implants, encapsulation (micro-, nano-, macro-), release modulator etc. (Dow, 2016, Murtaza, 2012, Dow, 2014, Rowe et al., 2009). This article therefore presents a scholarly survey of recent advances and future directions directly related to the functionalities of premium ethylcellulose polymeric biomaterials in the architecture of novel drug delivery systems intended for improving therapeutic and prophylactic outcomes. Key topics covered include ethylcellulose chemistry, synthesis, types, properties and recent developments on its application as an effective carrier for drugs and biologics.

## 2 Chemistry, synthesis and classification of ethylcellulose polymers

**Fig. 1:**
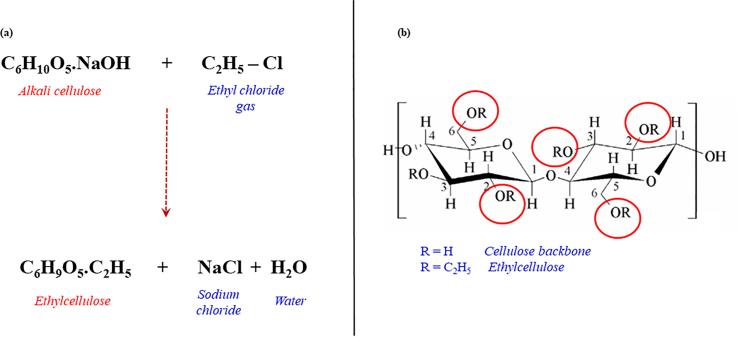
Illustrations of: (a) chemical synthesis of ethylcellulose (adapted from Rekhi and Jambhekar, 1995) and (b) chemical backbone structures of cellulose and ethylcellulose (adapted from Murtaza, 2012).

Ethylcellulose is a linear polysaccharide derived from the naturally occurring polymer, cellulose and therefore possesses its polymeric backbone which is based on the repeating structure of ß-anhydroglucose ring having three reactive hydroxyl functional groups (Davidovich-Pinhas et al., 2014, Rekhi and Jambhekar, 1995). The substitutable hydroxyl groups of cellulose can partially or completely react with chemical compounds or moieties to form derivatives possessing useful properties (Mahnaj et al., 2013). Ethylcellulose is synthesized through the substitution of the cellulose hydroxyl moieties with ethoxyl groups (Mehta et al., 2016). Synthesis (etherification) steps involved include the dissolution of cellulose in sodium hydroxide aqueous solution (50%^w^/_w_ or greater) leading to the breakdown of the cellulose supramolecular structure resulting in the formation of an alkali cellulose and exposure of the cellulose hydroxyl group for reaction. Subsequently, ethyl chloride gas is added to the reaction medium leading to reaction with the alkalized cellulose yielding ethylcellulose plus two by-products namely sodium chloride and water (Fig. 1) (Davidovich-Pinhas et al., 2014, Koch, 1937, Rekhi and Jambhekar, 1995).

The elicited physical characteristics of ethylcellulose polymer types and their performances are primarily dependent on the degree of etherification or substitution (ethoxyl content) and molecular weight of the cellulosic backbone (Davidovich-Pinhas et al., 2014, Koch, 1937, Mehta et al., 2016). For instance, water solubility is achieved with degree of substitution (DS) between the range of 1.0–1.5 while solubility in organic solvents is achieved with DS values in the range of 2.2–2.6 (Davidovich-Pinhas et al., 2014, Koch, 1937). Ethylcellulose commercial products usually have DS values ranging between 2.2 and 2.6 per anhydrous glucose unit and are marketed in a number of different viscosity grades. Usually, their viscosities increase as the polymeric backbone chain length increases and commercial grades are generally produced with differing viscosities while the ethoxyl content is strictly varied within standardized limits (Rekhi and Jambhekar, 1995, Mehta et al., 2016). The pharmacopeial specification provides allowance for variations between 80 and 120% within highlighted nominal viscosity while ethoxy substitution is fixed within a range of 44–51% (Mehta et al., 2016). The quality of the manufacturing processes plays a major role in ensuring that these set limits are met. For example, Dow Chemical Company, a key global manufacturer of premium ethylcellulose polymers marketed as Ethocel^TM^ employ strict measures and thereby have products with viscosity and ethoxyl substitution variations of 90–110% and 48–49.5% respectively (Table 1). Basically, six different ethylcellulose premium polymers are available specifically for pharmaceutical applications. These include Ethocel^TM^ standard 4, 7, 10, 20, 45 and100. In addition, standard Ethocel^TM^ 7, 10 and 100 premiums are available in the fine particle (FP) size also for pharmaceutical use (Table 1). The particle size and distribution can potentially influence their performance (e.g. drug release, compressibility etc.) in relation to their pharmaceutical application (Dow, 2014).

**Table 1:** Classes of commercially available premium ethylcellulose polymers (Dow, 2016, Rowe et al., 2009).

Premium Ethylcellulose Polymers (Ethocel™)	Solution viscosity (mPa.s)	Ethoxyl content (%^w^/_w_)	Maximum loss on drying (%^w^/_w_)	Particle size (μm)
4	3.0–5.5	48.0–49.5	2.0	N/A
7	6.0–8.0	48.0–49.5	2.0	N/A
7 FP	6.0–8.0	48.0–49.5	2.0	140.0 max, 5.0–15.0 mean
10	9.0–11.0	48.0–49.5	2.0	N/A
10 FP	9.0–11.0	48.0–49.5	2.0	100.0 max, 3.0–15.0 mean
20	18.0–22.0	48.0–49.5	2.0	N/A
45	41.0–49.0	48.0–49.5	2.0	N/A
100	90.0–110.0	48.0–49.5	2.0	N/A
100 FP	90.0–110.0	48.0–49.5	2.0	150.0 max, 30.0–60.0 mean

## 3 General properties in relation to pharmaceutical and biomedical usefulness

Premium ethylcellulose polymers (Ethocel™) can be described as white to light tan granular powders in physical appearance with bulk density and specific gravity of about 0.4 g/cm^3^ and 1.12–1.15 g/cm^3^ respectively (Dow, 2014, Rowe et al., 2009). Their glass transition temperatures range between 129 and 133 °C and they absorb minimal water from the atmosphere or even when inserted in water and the little absorbed water vapourizes readily (Rowe et al., 2009). Their attractive physical properties allow them to be used in a wide range of applications. They are considerably mechanically tough, thermoplastic, flexible (flexibility is retained when not plasticized and at temperatures as low as −40 °C), possess film forming properties, transparent which allows it application as coating agent in a variety of products (Davidovich-Pinhas et al., 2014, Koch, 1937). Their excellent thermoplastic nature and ability to soften at 135–160 °C makes them versatile in pharmaceutical hot melt extrusion processes which aids desired drug release properties and improved bioavailability of especially poorly soluble bioactives (Dow, 2016). They are thermally stable, have low flammability and can transmit light wavelengths between 2800 and 4000 Å (Koch, 1937).

Ethocel^TM^ polymers are inert to alkalis of all strength and dilute acids and demonstrates compatibility with a wide variety of organic materials such as waxes, resins and plasticizers (Koch, 1937). Their compatibility with organic materials have enhanced their use as film rheology modifiers, binding agents, adhesives and blends with other polymers and ceramic (Davidovich-Pinhas et al., 2014, Dumitriu, 2004). Ethylcellulose polymers are easy to use for solution applications because they are remarkably soluble a wide variety of solvents which include aromatic hydrocarbons, alcohols, ketones, chlorinated solvents esters amongst many others (Dow, 2014, Rekhi and Jambhekar, 1995, Rowe et al., 2006). They are mostly readily soluble in solvents that have cohesive energies or solubility parameters that are closely related to theirs which differs depending on the degree of substitution of the respective Ethocel^TM^ polymer (Burrell and Immergut, 1975, Rekhi and Jambhekar, 1995). They are practically insoluble in glycerin, propylene glycol and water. Those that contain more than 46.5% of ethoxy groups are freely soluble in chloroform, 95% ethanol ethyl acetate, methanol and toluene while those that have less than 46.5% of ethoxy groups are freely soluble in chloroform, methyl acetate, tetrahydrofuran and in aromatic hydrocarbons plus ethanol mixtures (Rowe et al., 2009).

Ethylcellulose polymers are excellent film-formers and binders and are most frequently applied as coats or matrices in controlled release solid dosage formulations (Murtaza, 2012, Dow, 2014). They usually form strong, hydrophobic and highly adhesive films which have also been used to improve tablet integrity, appearance and taste. They are versatile, often perform multiple functions in a single drug formulation and are characterized by very minimal batch-to-batch variations (Dow, 2014). They often provide flexible diffusion barriers whose properties can be changed by the amount of pore-forming agents used, film thickness, solvent type and molecular weight of the ethylcellulose. These polymers are compatible with commonly employed coating techniques such as pan and fluidized bed (Dow, 2014). Drug release kinetics through the ethylcellulose coatings is mostly controlled by the process of diffusion through the different layers. Research has shown that this is often a retarded process especially for formulations like tablets, capsules etc. with the exception of drug carriers characterized by larger surface areas such as pellets and granules in which cases, the release kinetics tend to be significantly more rapid. Besides, drug release from formulations such as ethylcellulose coated microcapsules may also be a function of the capsular wall thickness in addition to the surface area (Grund et al., 2014, Muschert et al., 2009, Rowe et al., 2009, Zhang et al., 2016a, Zhang et al., 2016b).

Solutions of ethylcellulose in aromatic hydrocarbons are usually highly viscous except for low concentration solutions. In contrast, ethylcellulose dissolved in ethanol or methanol produce lower viscosity solution with poor film forming capabilities for practical applications. A mixture of these two solvent categories are reported to yield ethylcellulose solutions typified by their low viscosities and good film forming strength. Lower molecular weight aliphatic esters and ketones on the other hand yield ethylcellulose solutions with relatively low viscosities that produce films with good strength and elongation properties (Rekhi and Jambhekar, 1995).

They are susceptible to oxidative degradation in the presence of sunlight or ultra violet light at increased temperatures. This is often averted by the use of light absorbing (230–340 nm) antioxidants and chemical additives. Best storage conditions are usually less than 32 °C (90°F) in a dry area away from all heat sources. They are combustible, should not be stored near oxidizing agents such as peroxided and are known to be incompatible with paraffin and microcrystalline waxes. Ethylcellulose powders may irritate the eye and appropriate personal protective equipment (PPE) should be worn for protection.

They are highly compatible, globally acceptable polymeric material and comply with specifications of a number of regulatory bodies governing use in food, packaging, paper, adhesives and as pharmaceutical additives (Dow, 1998, Dow, 2016, Murtaza, 2012). Their non-ionic and hydrophobic nature makes them non-reactive and are often described as inert in vivo and in vitro (Davidovich-Pinhas et al., 2014, Dow, 1998, Dow, 2014, Koch, 1937, Murtaza, 2012, Rekhi and Jambhekar, 1995). They exhibit good stability within pH 3–11 making them useful in both acidic and alkaline mixtures. They are essentially tasteless, odorless and non-caloric making them ideal candidates for pharmaceutical, food and personal care applications (Dow, 1998, Rekhi and Jambhekar, 1995). They are generally employed in oral and topical pharmaceutical formulation as well as food products. They are not metabolized following oral consumption and therefore does not usually add to body calories. They are often not recommended for use as excipients in parenteral products because they are not metabolized in the body and may be harmful to the kidneys. Irrespective, they are normally viewed as non-toxic, non-irritating and non-allergenic. The World Health Organization has not really specified an acceptable limit for daily intake because they are not considered to be harmful to health (Rowe et al., 2009). Table 2 presents a summary of the functions of ethylcellulose polymers in related drug delivery systems.

**Table 2:** Functions performed by Ethocel™ polymers in delivery systems.

Type of drug delivery system	Function of ethylcellulose premium polymers	References
Tablets, granules, pellets, microspheres, Varnishes, elastomers	Hydrophobic coating, taste masking, matrix former, binding agent, release extender	Grund et al., 2014, Mohamed et al., 2015, Czarnobaj et al., 2017, Hossain et al., 2017, Zhang et al., 2018, Yang et al., 2018
Emulsions, colloids, dispersions, gels, Transungal systems	Stability enhancer, viscosity and release modifier	Phaechamud and Mahadlek, 2015, Srichan and Phaechamud, 2017
Transdermal systems	Mechanical strengthener, extended release, cutaneous deposition, backing layer for prolonged release	Idrees et al., 2014, Nidhi et al., 2016
Nanostructures	Taste masking, release retardant, increased encapsulation	Pan-In et al., 2014, Rao and Bhingole, 2015, Balzus et al., 2017, Taki et al., 2017
Nanofiber	Filament former	Yu et al., 2015
Implants	Hydrophobicity promoter, mechanical strengthener	Zaher et al., 2015

The appropriateness of a formed polymer film is dependent upon its apparent viscosity which is defined as the exhibited viscosity of a particular polymer concentration dissolved in a specific solvent at a known temperature. With the ethylcellulose polymers, viscosity levels are controlled by the polymer chain length which are dependent on the degree of polymerization or number of anhydroglucose units during synthesis. Ethylcellulose polymers exhibit a high glass transitioning point of about 140 °C and this is also related to the degree of substitution with a minimum level occurring at 2.55 (i.e. 48.5% ethoxy content). Like all polymers, the mechanical properties of ethylcellulose is dependent on their molecular weight. Gradual increase in their molecular weight increases their mechanical strength until a critical point when no further increase is recordable. This usually occurs at molecular weight of 7–8 × 10^4^ in commercially available film-coating polymers like ethylcellulose (Rekhi and Jambhekar, 1995). Visible or ultraviolet light has no discoloring effect on ethylcellulose. If not stabilized, these polymers are sensitive to oxidative degradation in the presence of sunlight or ultraviolet light and at increased temperatures. Therefore, heat and light stabilizers are often added, particularly in unpigmented formulations. Their average shelf life is two years and the appropriate storage conditions for Ethocel^TM^ polymer should not exceed 32 °C (90°F) in a dry and heat free area (Dow, 1998, Rekhi and Jambhekar, 1995).

## 4 Micro-structured drug carriers constructed from premium ethylcellulose polymers

Ethylcellulose polymers are commonly applied as matrices or encapsulants for drug molecules to form micro-structured delivery systems which can be used as single entities, in injections, filled into capsule shell or incorporated into tablet matrices (Huang et al., 2016, Mahmoud et al., 2018, Murtaza, 2012). Their key functions are controlled release and taste masking and various processing techniques such as polymerization, spray drying, pan coating, fluid bed granulation, emulsification etc. are often employed (Dow, 1998, Murtaza, 2012, Stulzer et al., 2008, Zaman et al., 2018). Research has shown that ethylcellulose polymers have been incorporated within the design templates of various micro-configured delivery systems loaded with wide range pharmaceutical actives for application via different routes and pharmacotherapeutic purposes (Duarah et al., 2017, Duffau et al., 2018, Howick et al., 2018, Kasashima et al., 2016, Nair et al., 2018). Table 3 summarizes examples of novel ethylcellulose-based micro-structures.

**Table 3:** Different drug delivery applications of ethylcellulose-based micro-structures.

Micro-structured delivery systems	Type; Function of Ethylcellulose	Other additives; Preparation method	Pharmaceutical active/drug	Pharmacotherapeutic indication	References
Microparticle-in-orally disintegrating tablet	Ethylcellulose suspension (Surelease®); Drug release retardant	Lipid, magnesium aluminometasilicate; Melt adsorption	Tamsulosin hydrochloride	Sustained release	Cho et al., 2018
Microcapsule	Ethylcellulose 10, 20, 100 cP; Carrier	Polyethylene glycol, diethyl phthalate, triethyl citrate, petroleum ether, magnesium stearate; Emulsion solvent evaporation method	Propranolol hydrochloride	Matrix former and flexible release kinetics for different therapeutic applications	Afrasiabi Garekani et al., 2018
Microparticle	Ethocel™ standard 45; Coating	Chitosan, Sodium tripolyphosphate; Ionotropic gelation/spray drying coupled with coacervation/solvent displacement	Doxycycline hyclate	Controlled release mucoadhesive delivery systems for periodontal disease	Gjoseva et al., 2018
Occular microsponges	Ethylcellulose (degree of substitution 2.42–2.53); Plasticizer	Pluronic F-127, polyvinyl alcohol, toluene, dichloromethane, calcium chloride; *Quasi* emulsion solvent diffusion	Acetazolamide	Enhance therapeutic efficacy and reduction in systemic advers effect associated with oral acetazolamide	Obiedallah et al., 2018
Hollow microspheres	Ethylcellulose 10, 45 and 100 cP; Release extender	Polyvinyl pyrrolidone, ethanol, ether; Emulsification and vaccum drying	Felodipine	Improvement of oral bioavailability	Pi et al., 2018
Microparticle	Ethocel^TM^ standard 7; Microparticle wall material	Eudragit L100 and S100; Spray drying	Indole-3-aldehyde	Enteric coating for targetted postbiotic small intestine delivery	Puccetti et al., 2018
Microsponge	Standard ethylcellulose, Carrier system and release extender	Dichloromethane, polyvinyl alcohol, xanthan gum; Modified double emulsification technique	Tacrolimus	Sustained release and improved immunosupressant activity	Zaman et al., 2018
Microparticle-in-extended release oral flexible tablet	Standard ethylcellulose 10cP; Matrix former	Hypromellose; Granulation	Carbamazepine	Aid paediatric and geriatric compliance	Chandrasekaran and Kandasamy, 2017
Microsponges	Standard ethylcellulose; Plasticizer	Eudragit S100, triethylcitrate, polyvinyl alcohol, sodium carboxy cellulose, magnesium stearate; Q*uasi* emulsion solvent diffusion technique	Prednisolone	Colon specific drug delivery for efficacy enhancement	Kumari et al., 2018
Polymeric microspheres	Ethylcellulose 300 cP; Drug carrier	Eudragit L100-55;Oil-in-oil solvent evaporation technique	Ivabradine hydrochloride	pH dependent drug release kinetics was achieved	Majeed et al., 2017
Microsphere	Ethocel 20 cP; Release retardant	None; Precipitation and solvent evaporation	Repaglinide	Sustained drug release and improved antidiabetic effect	Okunlola et al., 2017
Microcapsule	Ethylcellulose 20 cP; Encapsulant	Polyethyleneglycol 6000, ethylacetate; Emulsion-solvent diffusion	Metoprolol succinate	Drug encapsulation efficiency was significantly increased.	Song et al., 2017
Microparticles loaded with a drug/salt/complex formulation	Aqueous dispersion of ethylcellulose (Aquaacoat® ECD); Coat and release retardant	Sodium lauryl sulfate; Spray drying	Mirabegron	Sustained release suspension for oral delivery minimizing side effects	Kasashima et al., 2016
Microparticles-in-textile-based drug delivery system	Ethylcellulose dispersion (Aquacoat® ECD), Release retardant	Propylene glycol, colloidal silicon dioxide, triethyl citrate; Spray drying	Naproxen	Wearable delivery system for sustained anti-inflammatory activity in patients who cannot take oral of parenteral treatment	Arici et al., 2014
Micromatrices	Ethocel™ 20 cP and Surelease; Sustained release, mechanical strengthener	Ethanol; Spray drying	Theophylline	Sustained release, improved absorption and bioavailability	Afrasiabi Garekani et al., 2015a, Afrasiabi Garekani et al., 2015b
Microparticle-in-platform	Ethylcellulose NF20; release control	None; Emulsification and solvent evaporation	Aminofluoride, sodium fluoride	Improve oral delivery of fluoride increasing controlled localized supply and protection against dental caries	de Francisco et al., 2013
Multifunctional layered microparticle	Ethocel™ 45 cP; Inert property, encapsulant	Ethanol; 3-fluid nozzle spray drying	Diclofenac sodium	Sustained release and protection of bioactive	Pabari et al., 2012
Dermal polymeric microparticles	Standard ethylcellulose (ethoxy 48–49.5%); Encapsulating excipient	Poly vinyl alcohol, poly (N-isopropylacrylamide), poly D,L lactide, poly D,L lactide coglycoside; Emulsification-solvent evaporation	L-Levothyroxine	Increased drug encapsulation characterized by minimal leakage and transdermal permeation enhancement	Azarbayjani et al., 2011
Microparticulated bioadhesive vaginal gel	Ethylcellulose 22 cP; Release decelerating polymer	Carbopol, hydropropylmethylcellulose, toluene, ethanol; Dispersion and solvent evaporation	Zidovudine	Prolonged antiretroviral activity and convenient for patient use	Chatterjee et al., 2011
Modified-release microparticle	Ethylcellulose 22 cP; Release extender	Cyclohexane, n-hexane, methanol; Phase separation by temperature transitions	Tizanidine and tramadol hydrochloride	Reduction of dosing frequency during adjuvant analgesic and muscle relaxant therapy	Aamir and Ahmad, 2010
Floating microparticles	Standard ethylcellulose; Floating enhancer	Ethanol, liquid paraffin, dichloromethane; Dispersion and solvent evaporation	Ranitidine hydrochloride	Extend release and gastroretention to improve the bioavailability of narrow window drugs	Mastiholimath et al., 2008

## 5 Nano-configured architectures based on premium ethylcellulose polymers

**Fig. 2:**
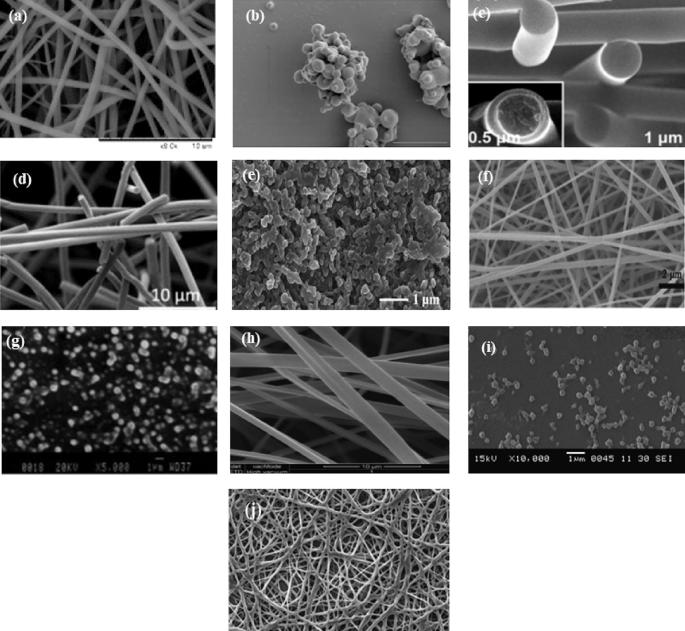
Select photomicrographs of ethylcellulose-based: (a) nanofibre hybrid mat delivery system (adapted from Li et al., 2018a, Li et al., 2018b), (b) nanofiber mesh system (adapted from Huang et al., 2012), (c) layered nanofiber delivery system (adapted from Yu et al., 2015), (d) amorphous monolithic nanofiber (adapted from Illangakoon et al., 2015), (e) nanocomposite (adapted from Taki et al., 2017), (f) composite nanofiber (adapted from Lu et al., 2017), (g) nanoparticles (adapted from Ravikumara and Madhusudhan, 2009), (h) polymeric nanofibers (adapted from Hu et al., 2016), (i) antibiotic nanoparticles (adapted from Pan-In et al., 2014) and (j) antibiotic nanocomposite (adapted from Djerafi et al., 2017).

Over the years, ethylocellulose polymers have been extensively applied in the development of bioactive loaded nano-structured delivery systems. Li and colleagues (2018) developed a ketoprofen-loaded dual-responsive nanofibre hybrid mat delivery system (Fig. 2(a)) based on a blend of ethylcellulose (6–9 mPa.s), poly (N-vinylcaprolactam) and Eudragit L100 using the twin-jet electrospinning technique. The developed nanosystem variants displayed uniformly distributed diameters ranging from 530 to 590 nm and they elicited both pH and thermal responsiveness as well as sustained release characteristics attributable to the hydrophobic nature of ethylcellulose. Besides, ethylcellulose was selected for this application because of its non-toxic, inert and hydrophobic properties (Li et al., 2018a, Li et al., 2018b). Likewise, Huang et al. (2012) designed ketoprofen-loaded ethylcellulose (6–9 mPa.s) based nanofiber mesh system that elicited a desirable biphasic controlled release behaviour (Fig. 2(b)). Ketoprofen containing zero order, layered nanofiber delivery systems (Fig. 2(c)) were also developed using triaxial electrospinning coupled with ethylcellulose 6–9 mPa.s functioning as a filament-forming matrix within the outer, middle and inner cores of the working solutions with varied adjuvant contents (Yu et al., 2015). Another study conducted by Illangakoon and team (2015) employed ethylcellulose 6–9 mPa.s as a 5-fluorouracil loaded core for the fabrication of an amorphous, monolithic fiber (Fig. 2(d)) by electrospinning.

The development of taste masked formulations is of importance in the pharmaceutical companies as this enhances patient compliance (Taki et al., 2017). The effectiveness of an ethylcellulose polymer (Ethocel™ 100 in the case) used in conjunction with other polmyeric materials as a taste-mask coating for very bitter Quinine, a known anti-malarial agent, was reported by Taki and group (2017). These formulations were developed in the form of nanocomposite particles (Fig. 2(e)) by spray drying using a two-solution mixing nozzle. In addition to the taste masking effect, the nanocomposite regulated the intestinal absorption of quinine (Taki et al., 2017) which was also partly due to the presence of an ethylcellulose polymer within the composite matrix. A study compared the dexamethasone release characteristics from ethylcellulose-based nanoparticles (Ethocel™ standard 4 premium) with other carriers namely Eudragit® RS100 and mixture of lipids. Overall, the EC nanoparticle display the most extended release behaviour (Balzus et al., 2016). In 2017, Balzus and co-workers also demonstrated that ethylcellulose improved the encapsulation efficiency/loading capacity of dexamethasone containing nanoparticles compared to Eudragit® while a combination of these two polymeric excipients yielded a drug-nanoparticulate matrix with maximized loading capacity, minimal toxicity, controlled dexamethasone release and mucosal permeation (Balzus et al., 2017). Besides, Rao and Bhingole (2015) applied the taste masking and slow release properties of ethylcellulose as a coating to the formulation of Gabapentin nanosponge-based controlled release, reconstitutable dry suspension for use in paediatric patients using the suspension layering technique.

The work of Lu et al., 2017 showed that the inclusion of ethylcellulose as a matrix former potentially improved mechanical properties of the entire delivery system. In this case, ethylcellulose was co-electrospun with zein (a key corn protein) to prepare a water-stable composite nanofibre formulation loaded with a non-steroidal antiinflammatory drug, indomethacin (Fig. 2(f)) which demonstrated sustained, diffusion-controlled release behaviour. Nimesulide release from ethylcellulose nanoparticles (Fig. 2(g)) prepared using the desolvation approach was slowed down and sustained relative to methylcellulose which was also tested under similar conditions. Heamatotoxicity studies also confirmed the compatibility of the ethylcellulose with blood cells attributable to the non-toxic nature of ethylcellulose polymers (Ravikumara and Madhusudhan, 2009). Similarly, Hu et al. (2016) demonstrated the biocompatible and extended release properties of ethylcelluose as it was employed in the architecture of polymeric nanofibers loaded with ketoprofen for tissue engineering and drug delivery applications (Fig. 2(h)). Amorphous nanocapsules of beclomethasone dipropionate were prepared for pulmonary delivery using ethylcellulose. Interestingly, these nanocapsules displayed encapsulation efficiency close to 100%, delayed photodegradation of pharmaceutical active, prolonged release without burst effect and insignificant cytotoxic effect (Chassot et al., 2015).

Nanoencapsulation of pharmaceutical actives using ethylcellulose has been shown to enhance antibacterial activity and minimize toxicity. Park and team reported the formulation of an antibiotic ethylcellulose nanofiber which demonstrated desirable antibiotic release trends with optimal and sustained antibacterial efficacy against Staphylococcus aureus as opposed to administering the antibiotic alone (Park et al., 2015). Encapsulation of clarithromycin into ethylcellulose (viscosity 250–300 cP, ethoxy content 48%) nanoparticles (Fig. 2(i)) for the treatment of four strains of *Helicobacter pylori* infection *in vivo* in murine models exhibited significantly improved antibacterial activity. Ethylcellulose encapsulation did not only enhance the anti-adhesion activity of clarithromycin but also significantly enhanced *H. pylori* clearance within the stomach of the infected murine model (Pan-In et al., 2014). Likewise, encapsulation of rifampicin with ethylcellulose (100 cP) was achieved using the supercritical anti-solvent process. Co-precipitation of ethylcellulose with rifampicin resulted in the formation of well encapsulated, drug loaded nanocomposites of diameters ranging between 190 and 230 nm (Fig. 2(j)). In addition to improving rifampicin’s encapsulation potential, its release was prolonged and bioavailability potentially improved (Djerafi et al., 2017). Furthermore, El-Habashy and co-workers (2016) investigated the potential of ethylcellulose-based nanoparticles in modulating the release kinetics and minimizing the ulcerogenic effect of piroxicam following oral administration. Nanoparticles were prepared in combination with other stabilizers added at different concentration using the evaporation technique and *in vivo* testing in rats showed a reduction in the mean ulcer index.

## 6 Macro-structured delivery systems fabricated from ethylcellulose polymers

Here, drug containing delivery systems or carriers with geometrical dimensions that fall outside the nano- and micro- meter ranges are discussed. These will include but not limited to tablets, films, capsules and phase transitional gels.

### 6.1 Diversified tablets

The oral route of drug administration remains the most preferred in patient care and tablets are the most common commercially available and acceptable oral preparation. Tablets with extended release characteristics are often more desirable especially for chronic conditions because they offer better patient compliance, consistent plasma drug levels, reduced dose (frequency and amount) and side effects as well as increased safety margin of high-potency drugs (Bejugam et al., 2015, Ravi et al., 2008). Considering the hydrophobic nature of ethylcellulose polymers, they have found extensive use in the fabrication of different drug-loaded tablets for improving the desired pharmacotherapeutic effects (Dow, 2014, Dow, 2016, Grund et al., 2014).

**Fig. 3:**
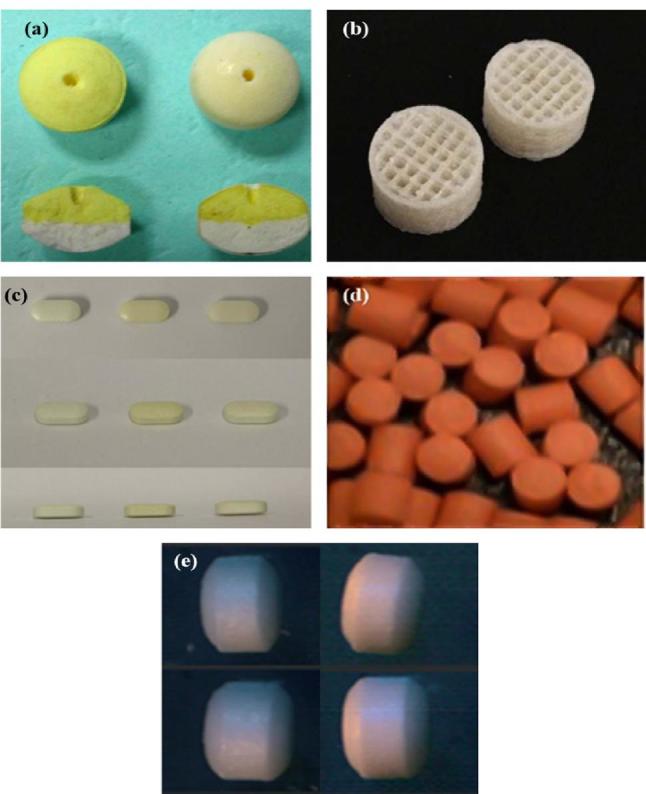
Ethocel™ polymer based: (a) osmotic tablet (adapted from Liu and Xu, 2008), (b) 3-D printed tablet (adapted from Yang et al., 2018), (c) gastroretentive tablet (adapted from Czarnocka and Alhnan, 2015), (d) mini-matrix tablets (adapted from Garber et al., 2015), (e) mini-tablet (adapted from Mohamed et al., 2015) dosage forms.

Studies have reported their use as semipermeable membranes either as single entities or in combination with other polymeric biomaterials in the preparation of osmotic tablets. They function by regulating the influx and efflux transfers of surrounding aqueous and drug molecules as well as control the disintegration kinetics of monolithic osmotic pump tablets (Ali et al., 2018, Li et al., 2012, Li et al., 2014, Liu and Wang, 2008), bilayer-core osmotic pump tablet (Liu and Wang, 2008) (Fig. 3(a)) and pulsatile core tablets (Lin et al., 2008). The have also been found to minimize burst effect, function as release retardants and hydrophobic coatings in elementary osmotic pump tablets (Alam et al., 2015, Arjun et al., 2016, Pan et al., 2017) and osmotic drug delivery systems (Patel et al., 2016) respectively.

Ethylcellulose polymers have found use as matrix formers and release extenders in oral tablet formulations (Mehsud et al., 2016, Patil et al., 2015). 3D printed tablets of ibuprofen with internal scaffolding structure (Fig. 3 (b)) elicited sustained release characteristics associated with the inclusion of ethylcellulose standard 10 which was first fused and then extruded in filaments with other excipients by hot melt extrusion. The resulting filaments were printed into extended release tablets by fused deposition modelling (Yang et al., 2018). In 2015, Nour and colleagues formulated an optimized microspheres in compressed tablet of bumadizone calcium dihydrate for colon targeting and controlled release. Their findings showed that microspheres based on ethylcellulose N100 displayed the highest entrapment efficiency which was attributed to its higher viscosity and ability to decrease drug diffusion to the outer phase increasing retention within the microsphere core as well as extended *in vitro* and *in vivo* release behaviour (Nour et al., 2015). The drug release retarding properties of ethylcellulose also found application in the construction of a once-daily dosing sustained release coated tablet of tolterodine-L-tartrate in the management of overactive bladder. This preparation compared well with the commercially available slow release formulation utilizing generated pharmacokinetic information (Pradhan et al., 2014). Caviglioli et al., 2013 developed diltiazem hydrochloride matrix tablets by direct compression coupled with thermal treatment and ethylcellulose N22 plus polycarbophil were employed as the matrix forming polymer. Tablet were stable and displayed a zero order prolonged release behaviour typified by anomalous transport mechanisms.

Research has shown ethylcellulose polymers to be valuable excipients for the construction of pulsatile tablets demonstrated by the work done by Shah and associates (2015) which detailed the optimization of a valsartan-loaded tablet designed to coordinate release dependent on the circadian rhythm. Ethylcellulose was shown to play a significant role in the modulation of the lag time and time taken for 90% of the drug to be released (Shah et al., 2015). This was also established by Patadia et al. (2016) where ethylcellulose-based coated-tablets of prednisone prepared by direct compression exhibited pulsatile release envisaged to facilitate the management of chronological disorders such as asthma. The efficacy of ethylcellulose as a release modulator was maintained but significantly influenced by the nature of other incorporated adjuvants. These pulsatile release tablets were further tested as carriers for other drugs namely methylprednisolone, diclofenac sodium, diltiazem hydrochloride, nifedipine and lornoxicam which displayed robust release trends (Patadia et al., 2017).

The versatility of the Ethocel™ polymers have also being displayed in their use as the only excipient or co-excipients in the development of specialty tablets designed for specific physiological target sites. Amongst these are the gastroretentive tablet matrices based on novel floating, high density, bioadhesive or swellable delivery technologies for improving the absorption and pharmacotherapeutic benefits of bioactive molecules (Bera et al., 2019). They have been utilized as release retardants (Meka et al., 2016), combination coats (Czarnocka and Alhnan, 2015) and matrix forming agents (Singh and Saini, 2016) in gastroretentive tablet formulations (Fig. 3(c)). Pathak and colleagues (2016) documented its performance as an effective mucoadhesive coat for the localized oral cavity delivery of fluconazole in a tablet preparation for oral thrush treatment. Another interesting contribution was that reported by Shah et al. (2016) and Kim et al. (2017) detailing their role, in conjunction with model Eudragit polymers, as coating and release sustaining agents in the design of colon-targeted delivery systems.

Mini-tablets are a single or multiple unit dosage form with a diameter of ≤6 mm composed of one or more excipients (Garber et al., 2015). Ethocel™ standard 100 premium was employed as part of the core component, coating and stability enhancer for a mini-tablet containing venflaxine hydrochloride. As a coating, Ethocel™ 100 extended the release of venflaxine from the mini-tablets but as a core component, it surprisingly enhanced rapid drug release which the authors attributed to polymer migration towards the surface and pore formation through the coat (Fig. 3(d)). Furthermore, a direct relationship between the thickness of the coat and prolonged venflaxine release capacity was observed for the minitablets (Garber et al., 2015). Ethylcellulose-based mini-tablets formulation loaded with theophylline were studied by Mohamed et al. (2015) (Fig. 3(e)). In this case, Surelease®, an aqueous ethylcellulose grade was employed as a film coating for the mini-tablets which displayed a prolonged theophylline release characteristics. Additionally, an increase in the Surelease® coating weight and a reduction in hydrophilic constituent levels within the mini-tablet matrices resulted in well-tailored, delay in lag and release times (Mohamed et al., 2015). Besides, ethylcellulose 100 cps was indicated in the construction of sustained release matrix type ocular minitablets containing timolol maleate for glaucoma treatment. Amongst other tested formulations, the ethylcellulose containing matrix was selected as the optimal preparation and it demonstrated a zero-order release kinetics with well-sustained timolol release patterns (Mortazavi et al., 2014). Recently, Li et al., 2018a, Li et al., 2018b also reported their work focused on employing ethylcellulose as a release-modifier for quetiapine fumarate containing mini-matrix prepared by hot melt extrusion.

### 6.2 Macrocapsules

Macrocapsules can also be referred to as capsules and they are often used as a macro-sized encapsulating shell for active pharmaceutical ingredients generally for oral administration. Different types of capsules with shells prepared from different biocompatible materials (e.g. gelatin, hypromellose) exist and are used for different purposes within the pharmaceutical industry (Biyani, 2017). Ethocel^TM^ polymers have been applied in different ways to enhance the performance of oral macrocapsules. An example of this is the work reported by Verma and colleagues (2014) whereby ethylcellulose was employed in the fabrication of cinnarizine-loaded films which were then folded in different patterns into hard gelatin capsule shell to form a compressed device for gastroretentive drug delivery. The polymeric film displayed an initial burst followed by a well-controlled non-Fickian diffusion pattern suitable for its intended application. Research has also shown the application of ethylcellulose as a body and cap in the development of an oral capsule delivery system which rapidly ejected incorporated payload of bioactives within the small intestine to improve bioavailability and efficacy (Waldner et al., 2014).

### 6.3 Transdermal and topical platforms

**Fig. 4:**
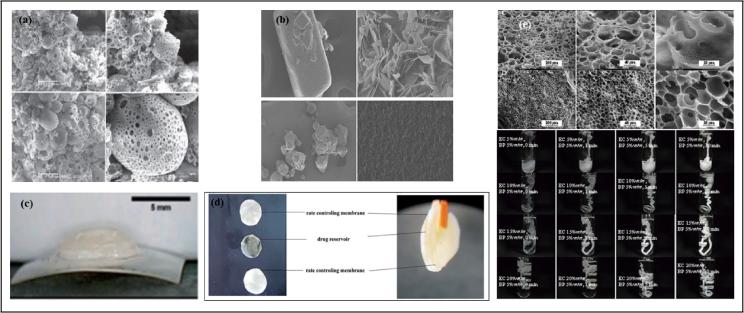
Ethylcellulose (a) microsponge gel for topical delivery (adapted from Bothiraja et al., 2014), (b) anesthetic and analgesic loaded transmucosal patch (adapted from Nidhi et al., 2016), (c) buccal disc for oromucosal drug delivery (adapted from Yildir et al., 2018), (d) timolol maleate-brimonidine tartrate ocular inserts (adapted from Ravindran et al., 2014), (e) in situ forming gel containing antimicrobials (adapted from Phaechamud and Mahadlek, 2015).

The skin extends over a large total surface area of the body which is approximately 1.8 m^2^ and functions as the main contact between the human body and its surroundings (Honeywell-Nguyen and Bouwstra, 2005). Delivery of drug molecules to the skin is mainly categorized as *dermal* referring to the process of mass transport of bioactives applied to the skin surface into various skin layers and the *transdermal* which encompasses the entire process of absorption of drug molecules from the skin surface (point of application) through each layer of the skin leading to their uptake by the skin microcirculation system and final distribution into the blood stream (Godin and Touitou, 2012, Honeywell-Nguyen and Bouwstra, 2005). Studies have detailed the effectiveness of ethylcellulose polymers as polymeric templates for drug delivery to the skin (Pattnaik et al., 2015). Bothiraja et al. (2014) confirmed this through their work focused on development of ethylcellulose microsponge gel for the topical delivery of eberconazole nitrate employing a quasiemulsion solvent diffusion method (Fig. 4(a)). The presence of ethylcellulose in the matrix facilitated controlled drug release and cutaneous deposition which aided non-toxic fungal therapy. Research reports have also documented the performance of ethylcellulose polymers in the presence of other adjuncts as matrix materials for transdermal patches designed for the systemic delivery lipophilic flurbiprofen (Idrees et al., 2014) and dexibuprofen (Akhlaq et al., 2016). In this case, the ethylcellulose patches variant showed the most desired permeation enhancing and flexible characteristics. Furthermore, a multidrug transmucosal drug delivery systems displaying complex release properties was developed by Nidhi and co-workers (2016) for use as a preoperative anesthetic and analgesic during dental surgical procedures (Fig. 4 (b)). Ethylcellulose viscosity 20 (50% ethoxy content) functioned as a backing layer for this system which demonstrated prolonged combined anesthetic and analgesic effects. Further enquiries by Roh et al. (2016) also confirmed the efficiency of ethylcellulose as a biocompatible backing layer for a mucoadhesive bi-layered strip for controlled delivery of lidocaine. Interestingly, Ethocel^TM^ polymers have also being indicated in the formulation of transdermal sprays for clotrimazole as an attempt to improve its *in vivo* antifungal performance (Paradkar et al., 2015).

### 6.4 Film constructs and discs

The utilization of ethylcellulose polymers alone or in conjunction with other adjuvants in the preparation of useful film-like drug carriers is well documented in recent times (de Toledo et al., 2016, Laffleur et al., 2018, Scoutaris et al., 2018, Yildir et al., 2018, Zhang et al., 2019, Zhu et al., 2018). Amongst other drug delivery applications are their use in the construction of buccal films for delivery within the oral cavity. This was evidenced through a study reported by Laffleur and team where ethylcellulose 10 cP was applied in combination with other pharmaceutical additives for the preparation of robust allantoin-loaded films for the management of dry mouth syndrome has been demonstrated. The findings showed that the produced films were functional, mucoadhesive, flexible and stable with great promise for treating various intraoral cavity diseases (Laffleur et al., 2018). A related study reported ethylcellulose functioning as a backing sheet for bilayered mucoadhesive buccal films containing a combination of ornidazole and dexamethasone prepared for treating oral ulcers. *In vivo and in vitro* observations showed positive effect on mucosal repair plus reduced ulcerations and inflammation (Zhang et al., 2018). In a similar way, fluticasone propionate was formulated for localized buccal delivery through a mucoadhesive buccal film formulation composed of ethylcellulose and other polymeric blends. Films based on ethylcellulose were selected as the best performing with desirable sustained release characteristics and potential therapeutic efficacy (Ammar et al., 2017).

Beyond buccal films, ethylcellulose-copovidone three layered thin oral film preparations containing three antihypertensive agents (hydrochlorothiazide, amiloride hydrochloride and carvediol) were printed using three course jet dispensing technology. The role of ethylcellulose as a release modifier was noticeable and the developed film can be used as personalized formulation for the delivery of combination drugs (Scoutaris et al., 2018). Building upon this, ethylcellulose in conjunction with *propolis* extract (BP) were developed into modified release films for the controlled liberation of metronidazole for the treatment of protozoa and anaerobic bacteria infections. Results showed that in addition to release modification, the film formulation protected the metronidazole molecules (de Toledo et al., 2016). Investigations directed towards their use as mucoadhesive buccal discs for oromucosal delivery (Fig. 4(c)) have also been recorded where ethylcellulose together with polyethylene glycol derivatives facilitated prolonged release of corticosteroids and/or anesthetics for effective treatment of recurrent aphthous stomatitis (Yildir et al., 2018). Furthermore, ethylcellulose together with Eudragit S100 and other hydrophilic polymers like polyvinyl alcohol were optimized and developed into biocompatible reservoir type triple-layered ocular inserts (Fig. 4(d)) for dual release timolol maleate and brimonidine tartrate intended for the management of glaucoma. Again, the ethylcellulose layer within the inserts served as the rate controlling membrane and extended drug release for up to 32 h *in vitro* (Ravindran et al., 2014).

### 6.5 Emulsions, colloids, dispersions and gels

In the formulation of semi-solid dispersions such as emulsions and gels, which are considered as fluid delivery systems, Ethocel™ polymers have been majorly applied as flow modifiers in the form of release extenders considering their water insoluble nature (Sallam et al., 2015). Ethylcellulose combined with glycerylmonooleate served as release sustaining carrier platforms for metronidazole in the form of mucoadhesive gels intended for periodontal application. Drug release followed the Fickian diffusion mechanism and a direct relationship between drug loading dose and release patterns was observed (Sallam et al., 2015). Other findings detail its function in the design of solvent exchanged *in situ* gel forming delivery system for antimicrobials (Fig. 4(e)) indicated in the treatment of periodontitis (Phaechamud and Mahadlek, 2015, Srichan and Phaechamud, 2017). Ethylcellulose played a major role controlling the viscosity of these systems and their abilities to exhibit optimized flow properties which directly influenced their sustained drug release behavior required for their intended periodontal applications (Phaechamud and Mahadlek, 2015).

Sun and colleagues reported the role of ethylcellulose as a matrix former in a Eudragit S100, castor oil, cremophor RH40, and 1,2-propylene glycol mix for the preparation of solid, micro-structured emulsified delivery systems containing osthole, a clinically applicable hydrophobic coumarin compound. Initially, a liquid osthole loaded self-microemulsifying delivery system was formed and this was transformed into a solid form through the application of the spherical crystallization technique. Interestingly, the more stable solidified micro-emulsified system retained its initial morphology, particle size and zeta potential as when in the liquid state and ethylcellulose remained functional as a matrix former and release extender evidenced through the *in vitro*/*in vivo* studies (Sun et al., 2018). 51% w/w ethoxyl content ethylcellulose nanodispersions were prepared using a facile method involving its dissolution in ethanol and subsequent dipping in xanthan gum solution as an anti-solvent. The nanodispersions functioned as stable, non-toxic effective stabilizers for drug loaded oil-in-water pickering emulsions (Wu et al., 2017). Another investigation by Bizmark and Ioannidis (2015) also reported the use of ethylcellulose colloidal nano-suspensions as stabilizers for foams and emulsions through mechanisms related to interfacial particulate interactions coupled with energy absorption and surface tension transitions. Overall, particulate stabilization of foams and emulsions has been the focus of several studies because they have been found to be more stable compared surfactant stabilized ones.

### 6.6 Beads, granules and pellets

**Fig. 5:**
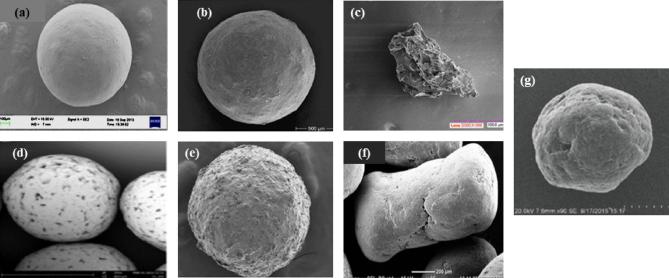
(a) Morphological differences exhibited by Ethocel™ based (a) ofloxacin-loaded floating bioadhesive multiparticulate delivery system (adapted from Zhang et al., 2016a, Zhang et al., 2016b), (b) multi-layered pellets (adapted from Muschert et al., 2009), (c) tacrolimus containing extended release granules (adapted from Tsunashima et al., 2016), (d) rough surfaced pellets (adapted from Priese et al., 2015), (e) osmotic theophylline pellets (adapted from Kazlauske et al., 2017), (f) reservoir type extended release multiparticulates (adapted from Franc et al., 2016), (g) wax based floating sustained-release dispersion pellets (adapted from Yan et al., 2016).

Researchers have also widely utilized ethylcellulose polymers as carrier templates for drugs and bioactives engineered in the form of nano- or micro-structured multiparticulates such as beads, granules and pellets (Hossain et al., 2017, Yan et al., 2016, Yang et al., 2014). As an ofloxacin-loaded floating bioadhesive multiparticulate delivery system (Fig. 5(a)), ethylcellulose in the form of Surelease® E-7-19040 combined with Eudragit® NE 30D were used to achieve optimal weight gain for robust pellet core structures and as coating which aided an extended release characteristic (Zhang et al., 2016a, Zhang et al., 2016b). Muschert et al. (2009) developed multi-layered pellet delivery systems (Fig. 5(b)) for different model drugs with different solubility values which aided comprehension of their release kinetics and mechanisms. Ethylcellulose aqueous dispersion NF (Aquacoat®) was employed as an external coat layer and it was discovered that amongst other applied excipients layers, it was dominant for exhibited sustained release characteristics, minimized potential effects of pellet core type and nature of the surrounding bulk fluid.

Czarnobai and team (2017) also presented their findings which explored ethylcellulose as a binding agent for the formulation of doxorubicin and metronidazole loaded bioactive mesoporous silica granular formulations useful for localized bone treatment and regeneration purposes. Key findings by Tsunashima and colleagues (2016) documented the performance of ethylcellulose standard 10 FP, as a release extender and solubility enhancer for poorly water-soluble tacrolimus formulated as extended release granules (Fig. 5(c)) intended to potentially prevent allograft rejection through *in vitro* investigations. *In vivo* experiments confirmed these outcomes as a sustained absorption of tacrolimus was observed male cynomolgus monkeys. Another study further supports this finding as an ethylcellulose film layer/coating over the pellet formulation (Fig. 5(d)) slowed down drug release rate while an increase in the thickness of this layer increased the lag time even more (Priese et al., 2015). It was noted that the surfaces of the pellets were rough and polyvinylpyrrolidone was used as a co-excipient to reduce pellet surface roughness. Interestingly, ethylcellulose film formation was improved and led to a more pronounced retardation of drug release from the pellets. As a release sustainer and bioavailability enhancer, ethylcellulose derivatives have been indicated as key adjuncts in the architecture of various multiparticulate drug delivery species which include matrices of theophylline (Afrasiabi Garekani et al., 2015a), osmotic theophylline pellets (Fig. 5(e)) (Kazlauske et al., 2017), capsaicin matrix pellets (Zhang et al., 2016a), osmotically active glucose pellets (Fig. 5(f)) (Franc et al., 2016), metoprolol tartrate and acetaminophen reservoir type extended release dosage form (Mehta et al., 2016), steroid hormone composite pellets (Hossain et al., 2017), oral time-controlled release etodolac pellets (Zhang et al., 2018, Zhang et al., 2019), glipizide beads (Nguyen et al., 2014) and atenolol pellets (Maboos et al., 2018).

In addition, studies have documented the application of ethylcellulose-based multiparticulate carriers for the targeted delivery of bioactives for eliciting specific pharmacological effects. An example was the investigation reported by Xu and co-workers (2014) whereby colon adhesive pellets of 5-aminosalicyclic acid were prepared as an oral modified release delivery system to minimize the side effects associated with systemic absorption for the treatment of ulcerative colitis. In this case, Surelease®, an ethylcellulose variant functioned as an inner layer for waterproofing and extended release. Surelease® was also applied as a release rate modulator in pH sensitive pellets intended for effective colon-targeted delivery of capecitabine to avoid frequent high dosing and associated systemic adverse effects (Pandey et al., 2018). Another investigation employed ethylcellulose together with pectin as a coating for oral minispheres that regulated ciclosporin release kinetics within the small intestine, enhanced delivery and uptake in the colon of the porcine model employed indicating improved clinical advantages in the treatment of inflammatory conditions of the large intestines (Keohane et al., 2016). Yan et al. (2016) also presented a study focused on the development of wax based floating sustained-release dispersion pellets (Fig. 5(g)) containing protocatechuic acid for localized timed gastric residency to improve its bioavailability and desired therapeutic performance. Ethylcellulose 100 cP was used as the coating which resulted in efficient sustained drug release activity of 12 h based on non-Fickian transport mechanisms. Extended release ibuprofen enteric coated pellets were successfully designed for small intestine delivery and ethylcellulose served as a water impermeable sustained release membrane. Optimized pellets demonstrated prolonged release characteristics comparable to commercially available ibuprofen preparations both *in vitro* and *in vivo* in beagle dogs (Dong et al., 2018).

### 6.7 Implantable structures

**Fig. 6:**
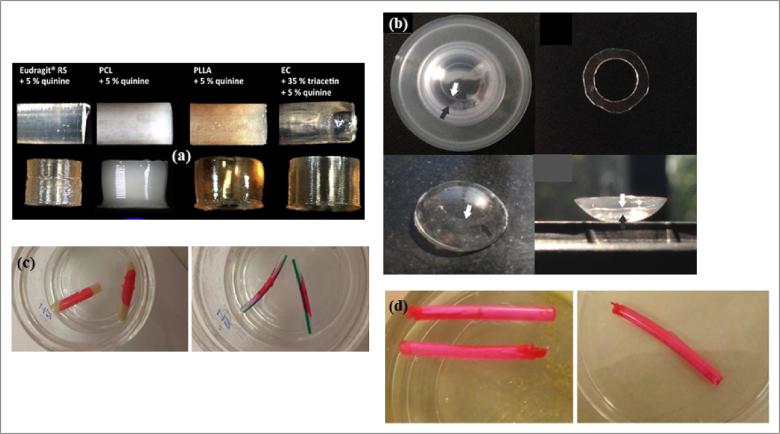
(a) Quinine loaded implantable matrix (adapted from Kempin et al., 2017), (b) hydrogel contact lenses for controlled drug delivery (adapted from Maulvi et al., 2016), (c) localized sustained release varnishes of thiazolidinedione-8, (adapted from Shenderovich et al., 2015) and (d) chlorhexidine sustained-release varnishes for catheter coating (adapted from Gefter et al., 2018) based on Ethocel™ polymeric derivatives.

Implantable structures are promising bioactive carriers that have been used for many biomedical purposes especially for the delivery of drugs that are not efficiently bioavailable when administered via the oral route or when site-specific dosing is important to patient care (Zaher et al., 2015). They are usually capable of providing long-term stability, controllability, biocompatibility and extended release kinetics with minimal side effects (Janićijević et al., 2019, Zaher et al., 2015). With Ethocel™ premium polymers being hydrophobic and mechanically strong in nature, they have found good use in the design of drug loaded implantable structures such as the osmotically driven remote-controlled magnetic nanocomposite membranes fabricated in 2015 by Zaher and colleagues. These membranes were based on either ethylcellulose or in some instances stronger cellulose acetate polymers mixed with thermosensitive poly (N-isopropylacrylamide) hydrogel and superparamagnetic iron oxide particles. The ethylcellulose containing prototype membrane devices had drug diffusion rates on the order 0.5–2.0 μg/h for higher release and 12–40 ng/h for lower release kinetics (Zaher et al., 2015). Ethocel™ standard 45 Premium showed desirable performance in the construction of quinine loaded implants Fig. 6(a) using the 3D printing technique of fused deposition modeling®. This work employed other polymers namely Eudragit®RS, polycaprolactone, poly(L-lactide) but the ethylcellulose based implant displayed the lowest prolonged release kinetics of 5% quinine over 100 days (Kempin et al., 2017)*.* Additionally, ethylcellulose 24–27 cP formed a major backbone for a timolol maleate loaded implantable nanoparticle-laden ring-in-hydrogel contact lenses (Fig. 6(b)) that was reported to provide controlled delivery kinetics at therapeutic rates without compromising critical contact lens properties for the management of glaucoma. *In vitro* release kinetic data showed sustained drug release within the therapeutic window for 168 h with a 150 μg timolol maleate loading. The implant was well tolerated displaying insignificant cytotoxicity and ocular irritation while *in vivo* pharmacokinetic studies in rabbit tear fluid indicated a substantial elevation of the mean residence time and area under curve with therapeutic efficacy comparable to conventional eye drop therapy. Furthermore, *in vivo* pharmacodynamic information showed a sustained reduction in the ocular pressure of a rabbit model for 192 h (Maulvi et al., 2016).

### 6.8 Sustained-release varnishes

Researchers have described varnishes as sustained release delivery systems that differ in their performances based on the nature of the polymer matrix, other pharmaceutical additives and therapeutic agents. They are capable of forming film structures which occur through physicochemical transitions within the polymeric chains and their mechanisms for controlled release are usually diffusion and erosion. Among the most widely used polymers for developing varnishes are the ethylcellulose and vinyl acetate-acrylate copolymers (Shapur et al., 2012, Shenderovich et al., 2015, Steinberg et al., 2002). Shenderovich and co-workers (2015) reported ethylcellulose N-100’s role in the development of local sustained release varnishes of an anti-quorum-sensing molecule, Thiazolidinedione-8, that has the potential to effectively prevent catheter-associated urinary tract infections, a major healthcare challenge. The thickness of the film and presence of other excipients were the main factors that affected drug release mechanisms based on the Higuchi model. The drug loaded ethylcellulose-based formulation displayed good retention for 8-days on both latex and silicon catheters and demonstrated activity against *Candida albicans* biofilms (Fig. 6(c)). A similar ethylcellulose N-100 varnish formulation designed for the prolonged delivery of chlorhexidine. They were particularly developed for application as catheter coatings for anti-biofilm activity of *Pseudomonas aeruginosa* (Fig. 6(d)). Chlorhexidine release was by diffusion and the varnish coating were retained on siliconized latex Foley catheters for at least 2 weeks and showed extended activity in a biological medium (Gefter et al., 2018). Ethylcellulose varnish formulations containing chlorhexidine were also applied to ureteral stents to minimize urinary tract bacterial colonization and proliferation on the stent surface. They effectively prolonged growth inhibition of *Enterococci*, *Pseudomonas*, and *Escherichia coli* for between 1 and 2 weeks (Zelichenko et al., 2013).

### 6.9 Elastomerics

Patients undergoing orthodontic treatment are usually prone to plaque build-up around their appliances and enamel demineralization around their brackets because oral hygiene is more difficult for these patients (Jeon et al., 2015). Consequently, Jeon et al., 2015, Jeon et al., 2017) applied extended drug release principles to improving delivery efficiency to the particular target sites. In 2015, they developed chlorhexidine-releasing elastomerics using ethylcellulose because of it proven safety, oral cavity efficacy and release extending qualities. An extended chlorhexidine release of 48 h and continued antibacterial effect on *streptococcus mutans* over this period was observed (Jeon et al., 2015). In 2017, Jeon and colleagues reported further performance enhancements for the extended release chlorhexidine elastomerics by applying a layer-by-layer approach using an ethylcellulose coat. Surprisingly, this extra ethyl cellulose coat significantly prolonged chlorhexidine release and antimicrobial efficacy for 7 days (Jeon et al., 2017).

### 6.10 Transungal delivery systems

Ethylcellulose has been indicated in the synthesis of a lipophilic matrix-based system of isotretinoin in the form of a nail lacquer intended for the treatment of nail psoriasis. This topical lacquer formulation was designed to form a film over the nail plate after the evaporation of solvent contained in the formulation. Drug delivery form the formed film was based on the existence of a drug concentration gradient that resulted in slow, penetrating release patterns through the nail plate. Ethylcellulose served as a biocompatible viscosity and release modifier for this formulation and compared well with commercially available Retino-A cream marketed preparation of 0.05%w/w isotretinoin. The nail lacquer was reported to show extensive distribution across the human nail plate model 72 h post application in comparison with the control formulation (Joshi et al., 2015).

## 7 Premium ethylcellulose polymers function as carriers for biologics and botanicals

Though not very common, Ethocel™ polymers have also being shown to function as robust platforms for loading and rate-modulated delivery of biologicals and botanicals. Such smart carrier systems are of different functional modalities ranging from nano- to micro- to macro- templates for different therapeutic uses (Howick et al., 2018, Ikeuchi-Takahashi et al., 2016). Standard ethylcellulose was used in combination with pectin, sodium alginate, calcium chloride, span 80 in the fabrication of enteric double coated microparticles of Biochanin A, a phytoestrogen isolated from *Trifolium pratense* that exert estrogenic effects and causes vasodilation and decrease in pro-inflammatory mediators, employing the ionotropic external gelation and coacervation phase separation coating method. Ethylcellulose was reported to aid release extension and improve oral bioavailability of poorly soluble Biochanin A by preventing stomach degradation (Sachdeva et al., 2016). Likewise, solid-lipid nanoparticles containing Morin (3,5,7,2′,4′-pentahydroxyflavone), a flavonoid from fruits, vegetables and tea were prepared from 49%w/w ethoxyl content Ethylcellulose 45 together with Witocan® H, Tween 60, polyvinyl alcohols using heated homogenization and evaporation. Ethylcellulose functioned as a release sustainer and overall, the formulation displayed improved oral bioavailability, aqueous solubility and minimized premature bioactive degradation (Ikeuchi-Takahashi et al., 2016). Still on botanicals, Sahu and colleagues (2013) developed ethylcellulose-based nanoparticles loaded with a plant-based flavonoid, Quercetin, for topical application in skin cancer chemotherapy utilizing the process of nanoprecipitation. In this formulation, ethylcellulose acted as a reservoir in skin ducts and furrows because of it biocompatibility and non-biodegradability and consequently aided bioactive retention and controlled release kinetics that can enhanced penetration through skin layers for treatment of skin cancer.

Additionally, biologicals such as L-alanyl-L-glutamine peptide effective in promoting acute glycemia recovery during long-term insulin-induced hypoglycemia have been encapsulated in standard premium ethylcellulose NF 20 microparticles by emulsification/hardening and solvent evaporation techniques. Ethylcellulose was shown to improve biocompatibility, formulation flexibility and overall prolonged peptide release to promote acute glycemia recovery and effective oral administration (Villa Nova et al., 2014). Howick and team (2018) encapsulated a dairy-derived hydrolysate, FHI-2571 know as a peptidic ghrelin agonist into pellet formulations made up of Ethocel™ standard 20 premium, methacrylic acid copolymer and microcrystalline cellulose utilizing dry blending combined with wet granulation, spheronization and drying. The presence of an ethylcellulose variant within the formulation mechanically strengthened the matrix, modified release kinetics and served as an external protective coat. Development of this formulation enabled oral dosing, *in vivo* performance and potential application in paediatric and geriatric patients (Howick et al., 2018). Moreover, Sharma and Sinha (2018) developed enteric coated nanosized emulsion capsules of cyclosporine A, an immunosuppressing cyclic polypeptide using Standard ethylcellulose, maize oil, oleic acid, Tween 20 and polyethylene gloycol 400 processed using homogenization coupled with macro-encapsulation. Ethylcellulose within this carrier system worked as a water insoluble membrane, primary/deep coating enhancing it’s colon targeting for better management of local intestinal pathologies.

## 8 Conclusions

Premium ethylcellulose polymers are biomaterials of noticeable value within the pharmaceutical and biomedical spheres. They function as excellent biocompatible excipients suitable for the architecture of a wide variety of active pharmaceutical ingredients which are transforming the face of medicines and improving human well-being globally. This has been evidenced through extensive *in vitro* and *in vivo* tests documented through scientific investigations as discussed. EthocelTM polymers are flexible and easy to process alone or with other excipients employing different re-fabrication techniques useful for the development of novel drug carriers. They usually function as release extenders, external coating, flexibility enhancers and mechanical strengtheners. They are typically tasteless, colorless, odorless, non-caloric and inert within the physiological environment, making them even more attractive for biomedical and pharmaceutical industrial applications. They are used as carriers for small drug molecules but enjoy limited use as delivery templates for botanicals and larger bioactive molecules like peptides. Considering their inertness, biocompatibility and stability at extreme temperatures, there is the need to expand their application towards the development of rate-modulated delivery systems for structurally larger biological molecules.

## Declaration of Competing Interest

None.
